# Tertiary drug information sources for treatment and prevention of COVID-19

**DOI:** 10.5195/jmla.2023.1662

**Published:** 2023-10-02

**Authors:** Robert D. Beckett, Yashawna Brattain, Judy Truong, Genevieve Engle

**Affiliations:** 1 robert.beckett@parkview.com, Clinical Standard Coordinator, Parkview Health, Fort Wayne, IN.; 2 YBrattain2024@manchester.edu, Manchester University College of Health Sciences and Pharmacy, Fort Wayne, IN.; 3 judytruong@creighton.edu, Drug Information Resident, Creighton University School of Pharmacy and Health Professions, Omaha, NE.; 4 genevieve.engle@belmont.edu, Director of the Drug Information Center and Associate Professor, Belmont University College of Pharmacy, Nashville, TN.

**Keywords:** Drug Information, Tertiary Databases, COVID-19

## Abstract

**Objective:**

To evaluate tertiary drug information databases in terms of scope, consistency of content, and completeness of COVID-19 drug information.

**Methods:**

Five electronic drug information databases: Clinical Pharmacology, Lexi-Drugs, AHFS DI (American Hospital Formulary Service Drug Information), eFacts and Comparisons, and Micromedex In-Depth Answers, were evaluated in this cross-sectional evaluation study, with data gathered from October 2021 through February 2022. Two study investigators independently extracted data (parallel extraction) from each resource. Descriptive statistics were primarily used to evaluate scope (i.e., whether the resource addresses use of the medication for treatment or prevention of COVID-19) and completeness of content (i.e., whether full information is provided related to the use of the medication for treatment or prevention of COVID-19) based on a 10-point scale. To analyze consistency among resources for scope, the Fleiss multi-rater kappa was used. To analyze consistency among resources for type of recommendation (i.e., in favor, insufficient evidence, against), a two-way mixed effects intraclass coefficient was calculated.

**Results:**

A total of 46 drug monographs, including 3 vaccination monographs, were evaluated. Use of the agents for treatment of COVID-19 was most frequently addressed in Lexi-Drugs (73.9%), followed by eFacts and Comparisons (71.7%), and Micromedex (54.3%). The highest overall median completeness score was held by AHFS DI followed by Micromedex, and Clinical Pharmacology. There was moderate consistency in terms of scope (kappa 0.490, 95% CI 0.399-0.581, p<0.001) and recommendations (intraclass correlation coefficient 0.518, 95% CI 0.385-0.651, p<0.001).

**Conclusion:**

Scope and completeness results varied by resource, with moderate consistency of content among resources.

## INTRODUCTION

The coronavirus disease 2019 (COVID-19) pandemic presented many challenges to healthcare providers. Knowledge of the biology and pathophysiology of SARSCoV-2 remains in its infancy, leading to frequent changes to recommended treatment options. With over 1 million US deaths since the onset of the pandemic, with a case-fatality ratio of 1.1%, the public health impact of COVID-19 remains high [1]. Since the beginning of the pandemic, the pressure to release information quickly and the global nature of the pandemic has challenged researchers' ability to conduct and complete rigorous, generalizable clinical trials [2, 3]. Thus, clinicians have often had to rely on the off-label use of drugs or await the release of study drugs for clinical use through the FDA Expanded Access Programs or Emergency Use Authorization. As of October 2022, 13 treatments for COVID-19 were being used off-label for emergency use while only 2 were FDA-approved [4]. However, repurposing drugs also demands a thorough process for determining the dose, treatment duration, and dosing regimen [5]. These parameters are conventionally studied in phase II clinical trials [6]. The absence of such trials for COVID-19 has made it difficult to establish cause and effect relationships and has further complicated the COVID-19 drug development process.

Especially during the early months of the COVID-19 pandemic, there was a lack of scientific evidence. This lack negatively impacted the public health response [7]. Tertiary, point-of-care, drug information databases are considered reliable sources of scientific evidence. We sought to evaluate these databases in terms of scope, consistency of content, and completeness of COVID-19 drug information. Specifically, we assessed the five major compendia, which are considered the most important online drug information resources: Clinical Pharmacology, Lexi-Drugs, AHFS DI (American Hospital Formulary Service Drug Information), eFacts and Comparisons, and Micromedex In-Depth Answers [8, 9, 10, 11, 12].

## METHODS

The five electronic drug information databases were evaluated in this cross-sectional evaluation study, with data gathered from October 2021 through February 2022. Study methods, including endpoints and statistics, were developed based on a similar previous study evaluating tertiary drug information database content for off-label uses [13]. The resources were selected based on the American Association of Colleges of Pharmacy (AACP) Basic Resources for Pharmacy Education list and have been included in previous studies [14, 15, 16, 17]. AHFS-DI was accessed through the Lexicomp database. A sample of medications was developed by extracting medications acknowledged by three major clinical practice guidelines: the National Institute for Health and Care Excellence (NICE), National Institutes of Health (NIH), and the Surviving Sepsis Campaign [18, 19, 20]. The medications did not need to be recommended in the guidelines to be included in the sample, in order to account for medications that had not been positively recommended in guidelines. To ensure that the list of drugs was as inclusive as possible, the sample was then reviewed by a subject matter expert in infectious diseases who recommended several additional medications.

Two study investigators independently extracted data from each of the five resources using a standard data collection form prepared in Microsoft® Excel®. A third investigator then verified and reviewed any divergence in the data through discussion with the original investigators. The five resources were searched using either the generic name of each medication from the pre-identified sample of agents, or the brand name if more than one brand was available for the same generic medication. Data were collected by searching through each drug monograph for information specifically related to COVID-19. In each resource, any information referencing indication (or lack thereof), dosage, route, and use in special populations (i.e., pediatrics, pregnancy, and geriatrics) for COVID-19 were extracted for review.

Descriptive statistics were primarily used to evaluate scope (i.e., whether the resource addresses use of the medication for treatment or prevention of COVID-19) and completeness (i.e., whether full information is provided related to use of the medication for treatment or prevention of COVID-19). For scope, the percentage of medications covered by the resource out of the total sample was calculated. For completeness, 1-point was awarded for presence of each of the ten listed completeness factors (itemized in Table 3) for each drug for a total possible score of 10 points. Aggregate results were described using median and interquartile range (IQR).

To group references into tiers according to scope and completeness, the highest scoring reference for each was compared to the next highest scoring reference, sequentially, until the difference was statistically significant using an alpha of 0.05. A sequential, tiers-based approach was selected to identify resources that had similar performance in each endpoint and to distinguish them from those resources that had higher or lower performance at each endpoint. Since the same sample of medications was evaluated in each resource, the McNemar test was used to compare scope (matched pair related categorical data) and the Wilcoxon signed-rank test was used to compare completeness (matched pair related ordinal data) [21]. To analyze consistency among resources for scope, the Fleiss multi-rater kappa was used. To analyze consistency among resources for type of recommendation (i.e., in favor, insufficient evidence, against), an intraclass coefficient was calculated using two-way mixed effects and measuring absolute agreement. Statistics were computed using IBM SPSS Statistics version 28.

## RESULTS

A total of 43 COVID-19 medications were identified from a review of clinical practice guidelines with 3 additional medications added following expert review (43 for treatment, 3 vaccinations, Table 1). As of October 2021, 40 of the 46 medications (87.0%) were acknowledged by NIH, 15 (32.6%) by NICE, and 10 (21.7%) by the Surviving Sepsis Campaign. Guidelines recommendations in favor of medication use were as follows: 16/40 (40.0%) in NIH, 11/15 (73.3%) in NICE, and 7/10 (70.0%) in Surviving Sepsis. However, NIH frequently recommended against use (14/40, 35.0%) or was indeterminate (10/40, 25.0%) whereas the other guidelines only included positive recommendations.

**Table 1:** Sample of medications acknowledged by COVID-19 clinical practice guidelines

Medications	NICE	NIH	Surviving Sepsis
Acalabrutinib		X	
Acetaminophen	X		X
Anakinra		X	
Atorvastatin		X	
Azithromycin	X	X	
Bamlanivimab/etesevimab			
Baricitinib		X	
Budesonide, inhaled		X	
Casirivimab/imdevimab		X	
Colchicine	X	X	
Convalescent plasma			X
COVID-19 vaccine, adenovirus	X	X	X
COVID-19 vaccine, mRNA, Moderna	X	X	X
COVID-19 vaccine, mRNA, Pfizer-BioNTech	X	X	X
Dexamethasone	X	X	X
Doxycycline	X		
Enoxaparin	X	X	X
Famotidine		X	
Fluvoxamine		X	
Hydrocortisone	X	X	
Hydroxychloroquine		X	X
Ibrutinib		X	
Interferon alfa		X	
Interferon beta		X	
Intravenous immunoglobulin		X	X
Ivermectin		X	
Lenzilumab		X	
Lopinavir/ritonavir		X	
Mavrilimumab		X	
Melatonin			
Methylprednisolone	X	X	
Nitazoxanide		X	
Otilimab		X	
Pravastatin		X	
Prednisone	X	X	
Remdesivir	X	X	X
Ruxolitinib		X	
Siltuximab		X	
Sotrovimab		X	
Thiamine			
Tocilizumab	X	X	
Tofacitinib		X	
Vitamin C		X	
Vitamin D	X	X	
Zanubrutinib		X	
Zinc		X	

NICE: National Institute for Health and Care Excellence; NIH: National Institutes of Health

In terms of scope, use of the agents for treatment of COVID-19 was most frequently addressed in Lexi-Drugs (n=34, 73.9%), followed by eFacts and Comparisons (n=33, 71.7%), Micromedex (n=25, 54.3%), Clinical Pharmacology (n=20, 43.5%), and AHFS DI (n=15, 32.6%) (Table 2). When grouped into tiers by scope, Tier 1 consisted of Lexi-Drugs and eFacts and Comparisons (p<0.05 vs. Tier 2), Tier 2 consisted of Micromedex and Clinical Pharmacology (p<0.01 vs. Tier 3), and Tier 3 consisted of AHFS DI.

**Table 2:** Scope results by resource

Medication	Clinical Pharmacology	Micromedex	Lexi-Drugs	AHFS-DI	eFacts and Comparisons
Acalabrutinib			X		X
Acetaminophen/paracetamol					
Anakinra	X	X	X		X
Atorvastatin					
Azithromycin	X	X	X	X	X
Bamlanivimab/Etesevimab	X	X	X	X	X
Baricitinib	X	X	X		X
Budesonide, inhaled			X		X
Casirivimab/imdevimab	X	X	X	X	X
Colchicine		X	X		X
Convalescent plasma		X	X		
COVID-19 vaccine, adenovirus	X	X	X	X	X
COVID-19 vaccine, mRNA, Moderna	X	X	X	X	X
COVID-19 vaccine, mRNA, Pfizer-BioNTech	X	X	X	X	X
Dexamethasone	X	X	X	X	X
Doxycycline					
Enoxaparin (for VTE prophylaxis)		X	X		X
Famotidine		X	X		X
Fluvoxamine		X	X		X
Hydrocortisone	X	X	X	X	X
Hydroxychloroquine	X	X	X	X	X
Ibrutinib			X		X
Interferon Alfa			X		X
Interferon Beta			X		X
Ivermectin		X	X		X
IVIG			X		X
Lenzilumab					
Lopinavir/Ritonavir	X	X	X	X	X
Mavrilimumab					
Melatonin					
Methylprednisolone	X	X	X	X	X
Nitazoxanide		X	X		X
Otilimab					
Pravastatin					
Prednisone	X		X	X	X
Remdesivir	X	X	X	X	X
Ruxolitinib	X		X		X
Siltuximab	X	X	X		X
Sotrovimab	X	X	X	X	X
Thiamine					
Tocilizumab	X	X	X	X	X
Tofacitinib	X		X		X
Vitamin C (ascorbic acid)		X			
Vitamin D					
Zanubrutinib			X		X
Zinc					
**Total:**	20, 43.5%	25, 54.3%	34, 73.9%	15, 32.6%	33, 71.7%

Table 3 describes the aggregate number of citations and individual completeness factors across medication entries, by resource. The highest overall median completeness score (1 point for each factor, up to a maximum of 10 points) was held by AHFS DI (6) followed by Micromedex (5), Clinical Pharmacology (5), eFacts and Comparisons (2.5), and Lexi-Drugs (2). When grouped into tiers by completeness, Tier 1 consisted of AHFS DI and Micromedex (p<0.05 vs. Tier 2), and Tier 2 consisted of Clinical Pharmacology, eFacts and Comparisons, and Lexi-Drugs.

**Table 3:** Completeness Factor Scores by Resource

n=number of COVID-19 drug monographs	Clinical Pharmacology (n=20)	Micromedex (n=25)	Lexi-Drugs (n=34)	AHFS DI (n=15)	eFacts and Comparisons (n=33)
**Provides a recommendation (n, %)**	20, 100%	21 84.0%	34, 100%	14, 93.3%	33, 100%
**Addresses regulatory status (n, %)**	20, 100%	20, 80.0%	8, 23.5%	10, 66.7%	33, 100%
**Cites a CPG (n, %)**	15, 75.0%	23, 92.0%	33, 97.1%	13, 86.7%	33, 100%
**Cites a clinical study (n, %)**	8, 40.0%	22, 88.0%	10, 29.4%	14, 93.3%	14, 42.4%
**Addresses statistical significance (n, %)**	1, 5.0%	14, 56.0%	1, 2.9%	4, 26.7%	0, 0%
**Provides an effect size (n, %)**	1, 5.0%	10, 40.0%	1, 2.9%	11, 73.3%	0, 0%
**Provides a specific dose (n, %)**	20, 100%	12, 48.0%	12, 35.3%	14, 93.3%	12, 36.4%
Addresses use in pediatrics (n, %)	11, 55.0%	6, 24.0%	8, 23.5%	7, 46.7%	9, 27.3%
Addresses use in pregnancy (n, %)	1, 5.0%	5, 20.0%	10, 29.4%	2, 13.3%	13, 39.4%
Addresses use in geriatrics (n, %)	4, 20.0%	0, 0%	0, 0%	2, 13.3%	1, 30%
**Total completeness score (median, IQR)**	5, 4.00-6.00	5, 3.25-6.75	2, 2.00-5.00	6, 5.00-7.00	3, 2.00-6.00

CPG: Clinical Practice Guideline

When consistency of scope was analyzed, there was moderate agreement (kappa 0.490, 95% CI 0.399-0.581, p<0.001) in coverage of the 46 medications among the 5 databases; as described above, inconsistency was driven by Lexi-Drugs and eFacts and Comparisons having greater coverage than other resources, especially AHFS DI. When consistency of content was analyzed using the type of recommendation as a surrogate, there was moderate agreement (intraclass correlation coefficient 0.518, 95% CI 0.385-0.651, p<0.001). The inconsistency appeared to be driven by Clinical Pharmacology, Micromedex, and AHFS DI being more likely to provide a recommendation in favor of use (100%, 81%, 86%, respectively) compared to Lexi-Drugs and eFacts and Comparisons (53% and 44%, respectively). The most commonly described medications, which were included in all 5 databases, were bamlanivimab/etesevimab, casirivimab/imdevimab, dexamethasone, hydrocortisone, hydroxychloroquine, lopinavir/ritonavir, methylprednisolone, remdesivir, sotrovimab, tocilizumab, and the 3 vaccinations. When considering the 65 recommendations for these 13 medications across the 5 resources, 57 (87.7%) of recommendations were positive, 4 (6.2%) were indeterminate, and 4 (6.2%) were against use.

## DISCUSSION

Overall, our study provided insight regarding the common drug information tertiary resources' scope, completeness, and consistency of content for COVID-19 drug information. Lexi-Drugs and eFacts and Comparisons were mostly likely to acknowledge or address the use of a medication for treatment or prevention of COVID-19, whereas AHFS DI and Micromedex provided more complete information. Consistency was reported as moderate agreement among all 5 databases. Specifically, Lexi-Drugs and eFacts and Comparisons together had greater coverage of the 46 COVID-19 medications (consistency of scope) and Clinical Pharmacology, Micromedex, and AHFS DI were more likely to recommend in favor of use (consistency of content).

Factors that contributed to low completeness scores included a lack of reported measures of statistical significance and effect size, and a lack of guidance for use in special populations, specifically geriatrics and pregnancy. More quantitative information from primary literature would help clinicians and health care providers make more nuanced treatment decisions that consider the clinical significance and risk-benefit ratio of the medication, not just whether the study was successful. These points may be helpful for information professionals to share with trainees, to help them identify when direct use of primary literature may be necessary to answer a clinical question. With many of these medications being new to market, it is expected that information guiding use in older adults and pregnant patients will emerge with time. In the meantime, with many COVID-19 clinical trials having substantial geriatric representation, the information provided for non-geriatric adults may be able to be extrapolated to geriatric patients.

Similar to the results of our study, Lexi-Drugs (Lexicomp) has been one of the top drug information tertiary resources in scope and consistency in a variety of analyses. In a study from 2007, Lexicomp was able to answer 82.9% of 158 drug information questions [15]. It is important to note that an update to this study would be an area for future research. Specifically, when assessing drug interactions in drugs of abuse, Lexicomp had the highest percent of interaction pair entries (43.4%) compared to the other databases [16]. In addition, the severity of interaction consistency was high in Lexicomp (83.1%). Similar scope score and consistency results were reported for drug-ethanol interactions: 84.9% and 75.6% respectively [17]. Lexicomp was also found to have the highest sensitivity and negative predictive value in regard to detecting oral oncolytic drug interactions [22]. In studies specifically assessing pharmacogenomic drug information, Lexicomp was found to include the most information for drugs assessed compared to the other compendia [23, 24]. The results of our study and previous studies, which have consistently reported Lexicomp as one of the top resources, can assist heath sciences librarians in determining whether the cost of Lexicomp is justifiable. When conducting a search for new COVID-19 treatment options, Lexicomp was found to be the best resource to consult first due to the high scope scores (73.9%).

In contrast, eFacts and Comparisons has not typically held high scope scores in previous studies (15.1-69.8%) [16, 17]. However, the findings of our study are not surprising since both databases are now owned by Wolters Kluwer and are increasingly becoming more visually and contextually similar. This finding also highlights that the quality and completeness of resources may vary over time and by subject matter.

When comparing our completeness results to other studies, Micromedex has also been consistently high. In the study from 2007, Micromedex had a completeness score of 97.0% [15]. In the studies assessing drug interactions in drugs of abuse and drug-ethanol interactions, Micromedex had completeness scores of 5 out of 5 in both cases [16, 17]. Most notably, a similar recent study found that Micromedex had among the highest scores for scope and completeness looking at information for off-label uses and infectious disease information [13, 25]. Conversely, compared to Clinical Pharmacology and Lexicomp, Micromedex reported fewer drug-drug interactions for assessed antiviral medications in a recent study [26]. These results, in combination with assessment of user needs and other comparable resources in the collection, will assist health sciences librarians in assessing the appropriateness of continued support of Micromedex despite price increases. Micromedex's completeness score makes this resource a critical second option to search when conducting COVID-19 research. When end users are seeking a resource that provides comprehensive information on off-label uses, this study and others suggest Micromedex may be a good option for information professionals to recommend.

To our knowledge, this is the first study of its kind to assess COVID-19 drug information scope, completeness, and consistency of content in commonly used databases. These results are important to inform practitioners of the best tertiary resources to consult for COVID-19 treatment information in this ever-evolving landscape and may also provide guidance in future pandemics or situations of rapidly evolving medication information. Additionally, health science librarians and drug information pharmacists would benefit from this contribution to the literature as it illustrates resources that provide well-referenced information during a health crisis and are most reliable when conducting research. When educating medical personnel in the use of resources, librarians can utilize the results of our study to illustrate the importance of checking more than one resource, given lack of consistency among resources. In addition, librarians can highlight Micromedex and AHFS as the best options for completeness of information and Lexicomp as the best resource in terms of scope for information related to COVID-19. When conducting a literature search for an end user, all resources provide helpful context, but Micromedex and AHFS appear to most consistently cite and discuss primary literature that can be used as a starting point.

Some strengths of our study include the thoughtful and rational approach for determining our treatment sample. Clinical practice guidelines were consulted to determine which COVID-19 medications should be searched in the databases. As a result, our sample was comprehensive at the time of generation and even included dietary supplements. All five major compendia databases were included in our study. Data collection was conducted by two independent investigators and discrepancies were assessed by a third investigator.

Our study had a few notable limitations. First, during the COVID-19 pandemic, information about these medications was constantly being updated. As a result, the information obtained from the resources may have changed since our analysis. AHFS-DI is updated monthly whereas Micromedex, Lexi-Drugs, eFacts and Comparisons, and Clinical Pharmacology are updated daily [27, 28, 29, 30]. This could have affected the performance of AHFS-DI in our study. The approach to assessing completeness of the information was used from previous studies but was not validated, though there is not currently a fitting, validated alternative. If a validated alternative becomes available, these resources should be reassessed using a validated measure. Additionally, differences in language were discovered in the databases. For example, some resources (e.g., Micromedex) tended to provide their own recommendations, based on the literature, whereas others simply summarized what was being stated in the literature without critical appraisal or assessment (e.g., Clinical Pharmacology, AHFS DI). Consistency of scope and recommendation results suggest that some databases (e.g., Clinical Pharmacology) may be more likely to acknowledge medications being used for COVID-19 when that use is recommended, versus addressing other medications that may be used despite low levels of evidence.

Overall, our study will assist healthcare providers in determining the best resources for COVID-19 treatment information and provide health science librarians with justification for resource funding. While no resource is perfect, Lexi-Drugs (scope) and AHFS (completeness) were found to provide the most information regarding medications used to treat COVID-19. Our study highlights the importance of always checking more than one resource when responding to a drug information question, especially during the COVID-19 pandemic. If only one resource is used, important information could be missed, and patient care could be affected.

## Data Availability

Original data are available upon request to the corresponding author.

## References

[1] Coronavirus Resource Center. Mortality Analyses [Internet]. Johns Hopkins University & Medicine; 2022 [cited 25 Oct 2022]. https://coronavirus.jhu.edu/data/mortality.

[2] Park JJH, Mogg R, Smith GE, Nakimuli-Mpungu E, Jehan F, Rayner CR, Condo J, Decloedt EH, Nachega JB, Reis G, Mills EJ. How COVID-19 has fundamentally changed clinical research in global health. Lancet Glob Health. 2021 May;9(5):e711-20. DOI: 10.1016/S2214-109X(20)30542-8. 33865476 PMC8049590

[3] Elkin ME, Zhu X. Understanding and predicting COVID-19 clinical trial completion vs. cessation. PLoS One. 2021 Jul;16(7):e0253789. DOI: 10.1371/journal.pone.0253789. 34252108 PMC8274906

[4] U.S. Food & Drug Administration. Coronavirus Treatment Acceleration Program (CTAP). The Administration; 2022 [cited 25 Oct 2022]. https://www.fda.gov/drugs/coronavirus-covid-19-drugs/coronavirus-treatment-acceleration-program-ctap.

[5] Shi J, Xiao Y, Zhang Y, Geng D, Cong D, Shi KX, Knapp RJ. Challenges of drug development during the COVID-10 pandemic: key considerations for clinical trial designs. Br J Clin Pharmacol. 2021 May;87(5):2170-85. DOI: 10.1111/bcp.14629. 33119136

[6] Clout AE, Pasqua OD, Hanna MG, Orlu M, Pitceathly RDS. Drug repurposing in neurological diseases: an integrated approach to reduce trial and error. J Neurol Neurosurg Psychiatry. 2019 Nov;90(11):1270-5. DOI: 10.1136/jnnp-2019-320879. 31171583

[7] Cornish L, Jerving S, Ravelo JL. Data around COVID-19 is a mess and here's why that matters [Internet]. Devex; 2020 [cited 25 Oct 2022]. <https://www.devex.com/news/data-around-covid-19-is-a-mess-and-here-s-why-that-matters-97077>.

[8] Clinical Pharmacology powered by ClinicalKey [Internet]. Elsevier; 2022 [cited 14 Oct 2022]. https://www.clinicalkey.com/pharmacology.

[9] Lexicomp®. Lexi-Drugs [Internet]. UpToDate, Inc.; 2022 [cited 14 Oct 2022]. http://online.lexi.com/lco/action/home.

[10] Lexicomp®. AHFS Drug Information [Internet]. American Society of Health System Pharmacists; 2022 [cited 14 Oct 2022]. http://online.lexi.com/lco/action/home.

[11] Lexicomp®. Facts and Comparisons [Internet]. UptoDate, Inc.; 2022 [cited 14 Oct 2022]. http://online.lexi.com/lco/action/home.

[12] IBM Micromedex® In-Depth Answers [Internet]. IBM Corporation; 2022 [cited 14 Oct 2022]. http://www.micromedexsolutions.com/micromedex2.

[13] Rothgeb A, Beckett RD, Daoud N. Off-label use information in electronic drug information resources. J Med Libr Assoc. 2022. Accepted manuscript, In-Press. 10.5195/jmla.2022.1419 PMC10124606 37101928

[14] Guy J, Portillo I, Beckett RD, Gill K, Mitchell K, Nissen J, Shenoy P. AACP Basic Resources for Pharmacy Education [Internet]. American Association of Colleges of Pharmacy; 2021 [cited 25 Oct 2022].

[15] Clauson KA, Marsh WA, Polen HH, Seamon M, Ortiz BI. Clinical decision support tools: analysis of online drug information databases. BMC Med Inform Decis Mak. 2007 Mar. 7:7. DOI: 10.1186/1472-6947-7-7. 17346336 PMC1831469

[16] Beckett RD, Martin JR, Stump CD, Dyer MA. Evaluation of drug information resources for interactions between therapeutic drugs and drugs of abuse. J Med Libr Assoc. 2020 Oct;108(4):584-590. 10.5195/jmla.2020.969. 33013215 PMC7524612

[17] Beckett RD, Stump CD, Dyer MA. Evaluation of drug information resources for drug-ethanol and drug-tobacco interactions. J Med Libr Assoc. 2019 Jan;107(1):62-71. 10.5195/jmla.2019.549. 30598650 PMC6300238

[18] National Institute for Health and Care Excellence. COVID-19 Rapid Guideline: Managing COVID-19 [Internet]. The Institute; 2021 [cited 25 Oct 2022]. https://www.nice.org.uk/guidance/ng191. 34181371

[19] National Institutes of Health. Coronavirus Disease 2019 (COVID-19) Treatment Guidelines [Internet]. The Institutes; 2022 [cited 25 Oct 2022]. https://www.covid19treatmentguidelines.nih.gov/. 34003615

[20] Surviving Sepsis Campaign. COVID-19 Guidelines [Internet]. The Campaign; 2020 [cited 25 Oct 2022]. https://www.sccm.org/SurvivingSepsisCampaign/Guidelines/COVID-19.

[21] DiCenzo R, ed. Clinical Pharmacist's Guide to Biostatistics and Literature Evaluation. 2nd ed. American College of Clinical Pharmacy; 2015.

[22] Marcath LA, Xi J, Hoylman EK, Kidwell KM, Kraft SL, Hertz DL. Comparison of nine tools for screening drug-drug interactions of oral oncolytics. J Oncol Pract. 2018 Jun;14(6):e368-e374. 10.1200/JOP.18.00086. 29787332 PMC9797246

[23] Chang JS, Pham DA, Dang MT, Lu Y, VanOsdol S, Shin J. Evaluation of popular drug information resources on clinically useful and actionable pharmacogenomic information. J Med Libr Assoc. 2016 Jan;104(1):58-61. 10.3163/1536-5050. 26807054 PMC4722644

[24] Vaughan KT, Scolaro KL, Anksorus HN, Roederer MW. An evaluation of pharmacogenomic information provided by five common drug information resources. J Med Libr Assoc. 2014 Jan;102(1):47-51. 10.3163/1536-5050.102.1.009. 24415919 PMC3878935

[25] Polen HH, Zapantis A, Clauson KA, Jebrock J, Paris M. Ability of online drug databases to assist in clinical decision-making with infectious disease therapies. BMC Infect Dis. 2008 Nov 6;8:153. 10.1186/1471-2334-8-153. 18990224 PMC2613899

[26] Drwiega EN, Badowski ME, Michienzi S. Antiretroviral drug-drug interactions: A comparison of online drug interaction databases. J Clin Pharm Ther. 2022 Oct;47(10):1720-1724. 10.1111/jcpt.13750. 36059105 PMC9826109

[27] AHFS Drug Information® and AHFS DI® Essentials™ [Internet]. American Society of Health System Pharmacists; 2023 [cited 4 April 2023]. https://www.ashp.org/products-and-services/database-licensing-and-integration/ahfs-drug-information-and-ahfs-di-essentials.

[28] Micromedex [Internet]. Merative; 2023 [cited 4 April 2023]. https://www.merative.com/clinical-decision-support.

[29] Editorial Process for Lexicomp [Internet]. Wolters Kluwer; 2023 [cited 5 April 2023]. https://www.wolterskluwer.com/en/solutions/lexicomp/about/content-editorial-process.

[30] Clinical Pharmacology Powered by ClinicalKey® [Internet]. Elsevier; 2023 [cited 5 April 2023]. https://elsevierresources.com/clinical-pharmacology-ck/.

